# Understanding the barriers to hiring autistic people as perceived by employers in the United Kingdom

**DOI:** 10.1177/13623613241301493

**Published:** 2024-12-04

**Authors:** Marianne Day, Chantelle Wood, Elizabeth Corker, Megan Freeth

**Affiliations:** The University of Sheffield, UK

**Keywords:** adults, autism spectrum disorders, vocational/labour force participation

## Abstract

Supporting more autistic people into employment is a major priority of the United Kingdom’s National Autism Strategy (2021–2026). However, little is known about the barriers employers perceive to hiring autistic people. A pre-registered cross-sectional survey study was conducted on a nationally representative sample of 1212 individuals with recent hiring experience. The Capability, Opportunity, Motivation – Behaviour framework and the Theoretical Domains Framework were used to identify perceived barriers to hiring autistic people. Results indicated that intentions to hire autistic people and to make adjustments to the hiring process were high. However, barriers existed in relation to knowledge about autism and how to make adjustments, as well as perceived limited opportunities to hire autistic people. Intentions to hire autistic people were higher for younger participants, men, people with higher levels of autism knowledge and those with more experience of previously hiring autistic people. Analysis of qualitative responses revealed employer concerns about workplace relationships, productivity and the need for training and adjustments to hiring processes. This study demonstrated that important targets for intervention are improving employer knowledge of autism and building more inclusive hiring practices. Practical suggestions for implementation are provided. These findings are significant for development of policies aimed at improving employment outcomes for autistic people.

**Lay abstract:**

In the United Kingdom, autistic adults struggle more to find jobs than non-autistic adults, which is a big concern to the government. To help more autistic people get jobs, hiring processes need to be better. By understanding what employers find challenging about hiring autistic people, we can come up with solutions to improve autistic people’s employment chances. A survey of 1212 employers and employees who hire people was conducted to find out what affects employers’ decisions to hire autistic people. Most people said they were open to hiring autistic applicants. This was particularly true for younger employers and men. People who had hired autistic people before were more likely to intend to do so again. However, key barriers to hiring were (1) not knowing enough about autism and (2) problems with how hiring is usually done in organisations. Employers also reported worrying about whether autistic employees would fit in at work, their productivity and the need for better training and changes to hiring methods. Our results indicate that it is important to educate employers about autism and make hiring practices more inclusive.

## Introduction

There are approximately 680,000 autistic adults living in the United Kingdom (Department of Work and Pensions (DWP), 2024). According to recent figures from the Labour Force Survey, only around 3 in 10 of autistic adults are in employment. The equivalent figure for disabled people overall is 5 in 10 versus 8 in 10 for non-disabled people. Further, employed autistic people are less likely to hold senior roles and they face the largest pay gap of all disability groups, suggesting that many in work are underemployed (Office for National Statistics (ONS), 2022). Employment provides opportunities for social interaction, financial independence and personal accomplishment (Evans & Repper, 2000) and has been associated with improved health and well-being in autistic people (Harkry, 2017). In addition, the unemployment and underemployment of autistic people have significant societal implications and economic consequences for employers, who are not fully accessing the skilled workforce available.

To address this pressing societal issue, the UK government has identified ‘supporting more autistic people into employment’ as one of six major priorities in the National Autism Strategy (GOV.UK, 2021). The strategy highlights the importance of supporting autistic adults to find and stay in work and enabling employers to be more confident in hiring and supporting autistic employees. In line with this priority, the Buckland review of autism and employment (DWP, 2024) provides recommendations aimed at reducing barriers to employment for autistic people. These include linking employers with appropriate advice on supporting autistic applicants, sharing positive recruitment stories with employers, highlighting the benefits of recruiting autistic people and providing models for sharing best practice. However, for barriers to genuinely be removed, it is necessary to understand what barriers employers perceive in relation to hiring autistic people. Although the Buckland review involved employer consultation in developing their recommendations as well as consulting with autistic people and specialist support groups, it did not include a large sample of employers, from businesses and organisations across all regions of the United Kingdom. A better understanding of employer knowledge, perspectives and practices is vital to design evidence-based solutions to improve employment outcomes for autistic people.

A broad range of research highlights the importance of making adjustments to hiring processes and the work environment to support the successful employment of autistic people (Annabi & Locke, 2019; Davies et al., 2023; Ohl et al., 2017; Rashid et al., 2018; Scott et al., 2017). If suitable adjustments are made, autistic employees can succeed and make substantial contributions in their workplaces (Tomczak et al., 2021). However, it is important to know where employers perceive barriers around these organisational processes and whether they feel they have the knowledge to develop solutions to overcome them.

Behavioural science frameworks provide a useful foundation for understanding and overcoming the potential barriers that hiring professionals might experience when hiring autistic people. The Capability, Opportunity, Motivation – Behaviour (COM-B) framework (Michie et al., 2013) combines numerous overlapping theories about the antecedents of behaviour. It proposes that for a person to perform a Behaviour (B) they require the Capability (C), Opportunity (O) and Motivation (M) to do so. Capability factors could be Psychological (e.g. knowledge about autism or workplace adjustments) or Physical (i.e. physical strength or skill, which is less relevant to hiring behaviours). Opportunity factors could be Physical (e.g. time and resources to support autistic employees) or Social (e.g. an organisational ethos which supports hiring an inclusive workforce). Motivation factors could be Reflective (e.g. positive or negative beliefs about hiring autistic people) or Automatic (e.g. feeling apprehensive about hiring autistic people).

The Theoretical Domains Framework (TDF; Cane et al., 2012) incorporates determinants of behaviour from 33 behavioural theories and consists of 14 more detailed domains that are nested within the six factors of the COM-B, allowing for a more nuanced understanding of behaviour. Specifically, knowledge, memory, attention and decision processes and behavioural regulation map onto the Psychological Capability factor of the COM-B model; physical skills map onto the Physical Capability; environmental context and resources are aspects of Physical Opportunity; social influences and social/professional role and identity map onto the Social Opportunity factor; reinforcement and emotions map onto the Automatic Motivation factor and beliefs about capabilities, optimism, intentions, goals and beliefs about consequences map onto the Reflective Motivation factor.

Together, these two models provide a comprehensive and complementary overview of the factors which underpin behaviour. Importantly, barriers identified using these frameworks can then be mapped directly onto intervention functions and techniques using the Behaviour Change Wheel tool, to suggest strategies for supporting positive behavioural change (Michie et al., 2014). For example, a lack of Psychological Capability in the form of gaps in relevant knowledge about autism could be addressed with educational strategies, while a lack of Physical Opportunity in the form of inadequate or non-inclusive hiring processes could be addressed via restructuring of the environment (i.e. implementing improved hiring processes and protocols). The TDF and COM-B frameworks have been shown to predict behaviours in different contexts, such as general healthcare and employment (e.g. Gibson Miller et al., 2020; Willmott et al., 2021). In a systematic review of the impact of COM-B factors on hiring outcomes for people with disabilities, Nagtegaal et al. (2023) found barriers across most of the COM-B dimensions, with the most frequent barriers being lack of knowledge and negative beliefs about productivity and the costs of hiring people with disabilities. However, the COM-B model has not previously been applied specifically to hiring autistic people.

The literature to date suggests that there are a number of barriers to hiring autistic people that map onto the COM-B framework. In terms of Psychological Capability, hirers may lack an understanding of autism and/or how to support autistic people in the workplace. For example, a stakeholder survey study found that lack of knowledge of autism was one of the main barriers to employment for autistic people, although this study only surveyed a small number of employers (35 of the 687 respondents were employers) and was instead based largely on the perspectives of autistic people and their families (Black et al., 2020).

Positive attitudes towards autistic people have also been shown to be associated with higher levels of knowledge about autism (Kim et al., 2023). Stereotyped and erroneous beliefs about autistic people may influence hiring behaviour, particularly in relation to Automatic and Reflective Motivation factors. Autistic people perceive negative stereotypes about autism in the workplace which reduces their likelihood of disclosing an autism diagnosis during the hiring process for fear of discrimination (Black et al., 2020; Davies et al., 2023). Likewise, studies show that employers hold negative beliefs about hiring autistic people. Employers assume that employing autistic people will increase operating costs and decrease productivity (Nagtegaal et al., 2023; Scott et al., 2017). They also have stereotyped perceptions about the work roles that autistic people are capable of which may make some job types less accessible to autistic workers (Andrade et al., 2022; Mai, 2019). Quantity and quality of previous contact with autistic people have been shown to be associated with more positive attitudes and reduced stereotypes towards autistic people (Dickter & Burk, 2021; Kim et al., 2023). This could be a facilitator for hiring outcomes but has not been explored specifically within a work context.

The need for sensitivity and awareness training for employers has been consistently highlighted as a target for reducing employment barriers, in terms of general awareness of autism (Annabi & Locke, 2019; Rashid et al., 2018), highlighting the challenges that autistic people face during hiring processes (Davies et al., 2023) and demonstrating how workplaces can be adapted to be more accessible to autistic people (Tomczak et al., 2021). However, much of the data this is based on are either from the perspective of autistic employees or small samples of employers. There is therefore a need to explore the need for educational input with a larger sample of employers from a range of organisation types and sizes.

Previous research has explored associations between demographic factors and attitudes towards autistic people. For example, in a meta-analysis exploring associations between non-autistic people’s attitudes towards autistic people and their personal characteristics, women were more likely to have positive attitudes towards autistic people, while age was not associated with attitudes (Kim et al., 2023). However, it is not known whether this would also apply in a workplace setting or what impact it might have on hiring intentions.

Employment outcomes for autistic people may also be influenced by organisational characteristics. In their systematic review of COM-B factors related to hiring disabled people, Nagtegaal et al. (2023) found that working for a larger organisation was a facilitator for hiring. Likewise, Scott et al. (2017) found that respondents from larger organisations were more likely to hire autistic people in a small sample of Australian employers and suggested that hirers from smaller organisations may perceive fewer available resources for supporting autistic people. Certain types of organisations have also been associated with greater motivations to hire autistic people. For example, the process of providing adjustments to attract autistic people has been described for particular industries such as large Information Technology (IT) companies (Annabi & Locke, 2019; Vogus & Taylor, 2018).

The existing literature shows that while there are some barriers which have been widely reported (e.g. lack of knowledge about autism, harmful stereotypes about autistic people), there are also less well-supported factors (e.g. demographic and organisational characteristics) which need further exploration. Some of these have not been explored in the context of employment, and more broadly, behavioural science frameworks have not been used to comprehensively map out barriers to hiring autistic people. There has also been less focus on employer perspectives and some studies have based their findings on small sample sizes. There is a clear need to systematically evaluate how these factors might relate together, and which are most impactful on hiring intentions. The present study aimed to assess these behavioural factors in a large sample of hirers from a range of business types and sizes, across the United Kingdom, using the COM-B and TDF frameworks. This will provide an evidence base to suggest the targets and behaviour change techniques best placed to effect real change on employment outcomes for autistic people.

## Method

Ethical approval for the study was obtained from the Research Ethics Committee of the Department of Psychology, The University of Sheffield. The study was pre-registered on the Open Science Framework (osf.io/x8tm3). Analyses described as exploratory were not included in the pre-registered protocol.

### Study design

This cross-sectional study used two online surveys administered using Qualtrics XM (2020): a screening survey to select the sample and a main survey to collect study data. The surveys were run between October and December 2023. Inclusion criteria were recent hiring experience (within the last 5 years) and residence in the United Kingdom. People who were autistic (either self or professionally diagnosed) were excluded, as barriers to hiring might be perceived differently by this group. Following the screening survey, an eligible sample was selected and invited to take part in the main survey.

A power analysis conducted in G Power, version 3.1 (Faul et al., 2007) indicated that 1099 participants were needed to detect small effects of *f*² = 0.02 (α = 0.05; Power = .95) in a multiple regression analysis with seven predictor variables. To account for attrition during the survey, and screening out of participants who did not pass the attention checks, the target sample size was set at 1200 participants.

### Participants

Participants were recruited via the online recruitment platform Prolific (www.prolific.com). Prolific filter questions were used to direct the screening survey to those who had hiring experience and were resident within the United Kingdom. A total of 2023 participants completed the screening survey. Participants (*n* = 388) were excluded if their hiring experience was not within the last 5 years or if they had a diagnosis of autism (either self-diagnosed or via a health professional), meaning that 1635 were then invited on to take part in the main survey on Prolific. After removing 16 participants’ data due to not passing all three included attention control questions (e.g. ‘If you are reading this question, please click agree’), the final sample was 1212 non-autistic participants with recent hiring experience. Participants were paid £4 for completing the study.

The demographic characteristics of the main sample are shown in Table 1. Mean age was 42.0 years (*SD* = 11.15) and the median age (40 years) matched the median age of the UK population (ONS, 2021a). There were more males in the sample (56.1%) in comparison to the UK population (49%; ONS, 2021a) and the sample was slightly higher in terms of ethnic populations from White (85.3%) and Black (6.6%) groups in comparison to the UK population (White: 81.7%; Black: 4%, ONS Census data, 2021b) and slightly lower for Asian (5.1%), Mixed (1.7%) and Arab (0.3%) ethnic groups in comparison to the UK population (Asian: 9.3%, Mixed: 2.9%, Arab: 2.1%, ONS, 2021b). Table 1 shows that the sample was drawn from across all regions of the United Kingdom, largely in line with the population census for 2021 (ONS, 2021b), with a slight skew towards a London population (19.6%) versus the UK population (13.2%). The largest industry groups were in the educational, health/social work, manufacturing, information/communication, financial/insurance and science/technology sectors.

**Table 1.** Participant characteristics of the main survey sample (*N* = 1212).

Participant characteristics		Count (% of sample)
Gender	Male	680 (56.1)
	Female	529 (43.6)
	Non-binary	2 (0.2)
	Not specified	1 (0.1)
Ethnicity	White	1034 (85.3)
	Black, Black British, Caribbean or African	80 (6.6)
	Asian or Asian British	62 (5.1)
	Mixed or multiple ethnic groups	21 (1.7)
	Arab	4 (0.3)
	Any other ethnic group	5 (0.4)
	Prefer not to say	6 (0.5)
Employment status	Full-time employee	942 (77.7)
	Part-time employee	124 (10.2)
	Self-employed	85 (7.0)
	Retired	41 (3.4)
	Unemployed	12 (1.0)
	Other	8 (0.7)
Main industry groups	Education	158 (13.0)
	Human health and social work activities	116 (9.6)
	Manufacturing	99 (8.2)
	Information and communication	94 (7.8)
	Financial and insurance activities	92 (7.6)
	Professional, scientific and technical activities	91 (7.5)
	Other	562 (46.4)
Business size	Large (250+ employees)	642 (53.0)
	Medium (50-249 employees)	260 (21.5)
	Small (11-49 employees)	195 (16.1)
	Micro (10 or less employees)	115 (9.5)
Geographical location	Greater London	237 (19.6)
	South East	159 (13.1)
	North West	147 (12.1)
	West Midlands	109 (9.0)
	East Midlands	91 (7.5)
	Yorkshire and the Humber	90 (7.4)
	South West	87 (7.2)
	East of England	86 (7.1)
	Scotland	82 (6.8)
	North East	59 (4.9)
	Northern Ireland	33 (2.7)
	Wales	32 (2.6)

### Measures

#### Screening survey

An initial screening survey collected personal and organisational demographic quantitative data: age, gender, ethnic group, industry type (using ONS industry classifications, ONS, 2021c), business size in terms of number of employees (following UK government guidelines, Department for Business, 2023) and location of business in the United Kingdom (using UK regions, ONS, 2021b). Participants were also asked for types of hiring experience (e.g. interviewing, writing job adverts, devising selection tasks) as well as frequency (e.g. once, twice) and recency of hiring experience (e.g. within the last year, 1–2 years ago). This survey was used to select a sample of participants with hiring experience within the last 5 years who were then invited to take part in the main survey.

#### Main survey

The main survey measured participants’: (1) current perceived and actual autism knowledge and perceived experience of autism, (2) intentions to hire autistic people and (3) factors related to hiring intentions (mapped to the COM-B model). The main survey mainly collected quantitative data, but optional open-ended questions were included to ensure that participants could raise barriers and issues not covered by the closed questions at the end of the survey. A full list of survey questions from each survey is included in the Supplemental Material and the included measures in the main survey are described in the following sections.

##### Knowledge of autism

Actual knowledge of autism was measured using the Autism Symptomatology Knowledge Assessment (ASKA: McMahon et al., 2020) and the Autism Stigma and Knowledge Questionnaire (ASK-Q: Harrison et al., 2017). The ASKA has 25 yes/no items that ask whether each symptom could be used to diagnose an individual with autism (e.g. ‘Inflexible adherence to routines’). ASK-Q has 49 items, answered using dichotomous agree or disagree responses (e.g. ‘I have heard of autism’). ASKA and ASK-Q total scores were calculated using their scoring rules and are scored out of a possible total of 25 for ASKA (McMahon et al., 2020) and 48 for the ASK-Q (Harrison et al., 2017).

Perceived knowledge of autism was measured using the total score from McMahon et al.’s (2020) 6-item questionnaire (5-point scale: 1 = Strongly Disagree to 5 = Strongly Agree; e.g. ‘I can recognise the signs and symptoms of autism’). Negatively worded items were reverse scored (e.g. ‘I would have difficulty explaining autism to someone else’). The total possible score for this scale is 30 (McMahon et al., 2020).

##### Experience of autism

Experience of autism was measured using questions designed for this study: (1) Does your company/organisation employ autistic people (yes, no, don’t know)? (2) I am experienced in hiring autistic people (5-point scale: 1 = Strongly Disagree to 5 = Strongly Agree), (3) As far as you are aware, have you previously worked with an autistic person (yes, no, don’t know)? (4) Have you received workplace training on autism (yes, no, not sure)? (5) Are any of your close contacts autistic (e.g. friends, family, colleagues)?

##### COM-B items related to hiring behaviours

Factors related to the hirer’s Capability, Opportunity and Motivation to hire autistic people (COM-B items) were measured using 43 items (5-point Likert-type scale: 1 = Strongly Disagree to 5 = Strongly Agree). These items were identified by the study authors from existing literature around employment barriers for autistic people and covered relevant domains of the TDF (Cane et al., 2012), which are nested under the COM-B framework (Michie et al., 2011). For example, items tapped into Psychological Capability (e.g. ‘I know enough about autism to hire autistic people’: TDF domain of Knowledge), Physical Opportunity (e.g. ‘Our organisation has policies and procedures in place to support autistic employees to work effectively’: TDF domain of Environmental context and resources), Social Opportunity (e.g. ‘Employing a diverse workforce is part of our organisation’s ethos’: TDF domain of Social influences), Automatic Motivation (e.g. ‘I feel positive about hiring autistic people to work in my organisation’: TDF domain of Emotion) and Reflective Motivation (e.g. ‘Supporting autistic employees in the organisation would cost my organisation too much money’: TDF domain of Beliefs about consequences). No items were included for Physical Capability (i.e. physical strength, skill and stamina) as this was not thought relevant in the hiring context. Participants were instructed to respond to these items in relation to the organisation where they had their most recent hiring experience (which could be in a previous role). This questioning format was chosen to encourage participants to reflect directly on their hiring experience rather than considering hypothetical situations which would be less likely to identify actual hiring barriers. A full list of the COM-B items is shown in Supplemental Table 1.

##### Intentions to hire autistic people

Intentions to hire autistic people were measured using 3 items: (1) ‘I intend to hire autistic people’, (2) ‘I am willing to hire autistic people’ and (3) ‘I will hire autistic people in the future’ (5-point scale: 1 = Strongly Disagree to 5 = Strongly Agree). A mean score was calculated which was used as the dependent variable in the regression analysis. Internal consistency for this scale was acceptable (Cronbach’s α = 0.86).

##### Optional open-ended questions

At the end of the survey, participants were asked four optional open-ended questions to allow for the identification of any further barriers to hiring autistic people: (1) What do you think would be the advantages of hiring autistic people? (2) What do you think would be the disadvantages of hiring autistic people? (3) Are there any other factors, which haven’t been mentioned in this survey, that make it difficult for your organisation to hire autistic people? and (4) Are there any other factors, which have not been mentioned in this survey, that would make it easier for your organisation to hire autistic people? This provided qualitative data to supplement the quantitative data on barriers to hiring autistic people.

### Data analysis

Means and standard deviations were calculated for the actual knowledge scales (ASKA and ASK-Q) to allow comparisons to be made with existing sample data (Harrison et al., 2023; McMahon et al., 2020). Linear regressions were also run to find whether actual knowledge of autism was predicted by perceived knowledge. Multiple linear regression was used to explore whether intentions to hire autistic people were predicted by age, gender, business size, previous experience of hiring autistic people, perceived knowledge and/or actual knowledge of autism.

Means and standard deviations were calculated for the COM-B items and were used to identify the most commonly perceived barriers to hiring autistic people. In addition, correlations between COM-B item scores and the scaled intention measure were calculated to identify which COM-B items were most closely associated with intentions to hire autistic people. All analyses of quantitative data were conducted using SPSS (version 29, IBM Corp., 2023).

Responses to the four optional, open-ended questions were analysed using an interpretive content analysis approach (Drisko & Maschi, 2015) which allows datasets to be described and summarised using an inductive, inferential approach. Participant responses were collected under the four question theme headings: (1) advantages and (2) disadvantages of hiring autistic people, (3) barriers and (4) facilitators for hiring autistic people, using separate Excel (Microsoft, 2019) spreadsheets. Each response was initially coded by MRD, with additional codes added iteratively until all the responses had been included. The coding lists for each theme were checked and agreed by other members of the research team (CW, MF). The whole dataset was then recoded with these coding frames. Finally, the number of times each code was mentioned was counted to give a measure of how commonly specific advantages, disadvantages, barriers and facilitators were mentioned by respondents. The coding frameworks including themes and codes can be seen in Supplemental Table 2 which also reports frequencies of responses.

### Community involvement

The project was overseen by a steering group which consisted of seven autistic adults alongside members of the research team and the Sheffield Occupational Health Advisory Service. The steering group advised on the content of the surveys and gave insights from their own lived experiences. They also discussed how the results of the survey could be applied to changes in hiring practices to reduce barriers within the workplace and how these results should be disseminated.

## Results

### Understanding the knowledge base of participants

The mean score on the ASKA scale in the current sample of UK-based employers and employees with hiring experience was 18.55 (*SD* = 2.75). The mean score for ASK-Q was 41.37 (*SD* = 3.17). These scores were comparable to general population samples in the United States and Canada (Harrison et al., 2023; McMahon et al., 2020). To examine whether perceived knowledge of autism is associated with actual knowledge of autism, separate linear regressions were run for the two actual knowledge scales (ASK-Q and ASKA). These analyses found that perceived knowledge was not associated with actual knowledge on either scale (ASK-Q: adjusted *R*^2^ = 0.00, *F* (1,1210) = 0.470, *p* = 0.493; ASKA: adjusted *R*^2^ = 0.00, *F* (1,1210) = 1.497, *p* *=* 0.221).

### Understanding the factors associated with intentions to hire

Multiple linear regression was used to examine whether (1) age (in years); (2) gender (male/female); (3) business size (Department for Business, 2023); (4) perceived experience of hiring autistic people; (5) total score on Perceived Autism Knowledge scale (McMahon et al., 2020); total score on actual autism knowledge scales; (6) ASKA (McMahon et al., 2020) and (7) ASK-Q (Harrison et al., 2017) were associated with intentions to hire autistic people. While we pre-registered a plan to combine ASKA and ASK-Q scores into a single actual knowledge scale, the scores on these two scales were only weakly correlated with each other (Pearson’s *r* = .27, *p* ⩽ 0.001) hence combining was not deemed appropriate. As such, we retained the ASKA and ASK-Q as separate scales in the regression. As there were four levels of business size, these were dummy coded into three variables (with large business size as the reference category) to enable this factor to be entered into the regression analysis. To allow the overall effect of business size to be assessed, these three variables were added as a second step of the regression and the change in *R*^2^ was assessed.

The first step of the regression model (age, gender, perceived hiring experience, perceived and actual autism knowledge) was statistically significant (adjusted *R*^2^ = .21, *F* (6,1205) = 52.93, *p* ⩽ 0.001) indicating that the specified factors explain 21% of the variance in intentions to hire autistic people. The change in *R*^2^ resulting from adding in business size in a second step in the regression model was statistically significant (adjusted *R*^2^ = .21, *F* change (3,1205) = 3.56, *p* = 0.014) although the model with business size only explained an additional 0.5% of the variance.

Table 2 shows the beta coefficients for the regression model and demonstrates that higher levels of perceived hiring experience, perceived knowledge and actual knowledge measured using the ASK-Q were associated with stronger intentions to hire autistic people. Perceived hiring experience accounted for the largest amount of variance in intentions to hire autistic people. In contrast, actual knowledge measured using the ASKA was not significantly associated with intentions to hire autistic people. Younger age and male gender were also associated with stronger intentions to hire.

**Table 2.** Results from the multiple regression of predictive variables on intentions/willingness to hire autistic people.

Predictor variable	Standardised beta co-efficient	*t*	*p*
Perceived knowledge	.059	2.04	0.04
Actual knowledge (ASK-Q)	.089	3.28	0.001
Actual knowledge (ASKA)	.002	0.63	0.95
Previous hiring experience	.380	13.34	<0.001
Age	−.130	−4.96	<0.001
Gender	.082	3.13	0.002

### Understanding the perceived barriers experienced by hirers around employing autistic people

To explore the perceived barriers to hiring autistic people, means and standard deviations for scores on the 43 COM-B items were compared (see Supplemental Table 1 for the full list of items). Items were generally worded in a positive direction (e.g. ‘I know how to make the hiring process more accessible for autistic people’), such that a lower score indicates that the relevant item posed more of a barrier to hiring autistic people. To ensure that means for each question could be interpreted in the same way, negatively worded items (e.g. ‘Hiring autistic people would decrease my organisation’s performance’) were reverse scored, so that a lower score similarly indicated that the relevant item posed more of a barrier. Reverse-scored items are indicated by an asterisk.

Mean scores were used to identify the most commonly experienced barriers. Table 3 shows the most frequently experienced barriers which are related to Psychological Capability (lack of knowledge and skills) and Physical Opportunity (particularly around current hiring processes) factors. Key barriers to hiring autistic people were lack of knowledge of autism and relevant employment law, lack of knowledge about how to make adjustments to hiring processes and work environments and a lack of existing systems and processes for making and monitoring adjustments. Participants demonstrated awareness that they would need to change their current hiring practices and the working environment to make these more accessible for autistic people.

**Table 3.** Lowest scoring means on the COM-B items, representing key barriers to hiring autistic people. Mean scores for COM-B questions (scored from 1 = Strongly Disagree to 5 = Strongly Agree).

COM-B item	COM-B domain	Mean (*SD*)
I use strategies to monitor how well I adjust hiring practices (e.g. job adverts, interview processes) for autistic people	Psychological Capability	2.72 (1.13)
I would need to change the way I work, to make adjustments to the working environment for autistic people^a^	Psychological Capability	2.72 (1.14)
I know how to make the hiring process more accessible for autistic people	Psychological Capability	2.81 (1.17)
I would need to change my current hiring practices to be able to hire autistic people^a^	Psychological Capability	2.84 (1.18)
I know about relevant employment law in relation to employing autistic people	Psychological Capability	2.85 (1.26)
During the hiring process, I consider whether the applicant could be autistic	Psychological Capability	2.92 (1.16)
I know enough about autism to hire autistic people	Psychological Capability	3.01 (1.16)
Our current hiring processes could be improved to enable autistic people to perform well^a^	Physical Opportunity	2.08 (0.87)
My organisation has systems and strategies to monitor whether adjustments to hiring processes (e.g. to job adverts, interview processes) are being made for autistic people	Physical Opportunity	2.89 (1.22)
My organisation has processes for making adjustments for autistic people during the hiring process (e.g. to job adverts, interview processes)	Physical Opportunity	3.03 (1.25)

a Scores for this item have been reverse scored.

Table 4 shows the COM-B items with the highest mean scores. Scores on Reflective and Automatic Motivation factors indicated that participants were generally motivated to hire autistic people, had high willingness to adjust hiring processes and the working environment, had positive beliefs about the capabilities and contributions of autistic people within the workforce and had positive emotions related to employing autistic people. High-scoring Social Opportunity factors included support for a diverse workforce in the ethos of their organisation and from colleagues and service users.

**Table 4.** Highest scoring means for the COM-B items, representing factors which were supportive of hiring autistic people. Mean scores for COM-B questions (scored from 1 = Strongly Disagree to 5 = Strongly Agree).

Com-B item	COM-B domain	Mean (*SD*)
If it led to better performance in my organisation, making adjustments for autistic employees would be worthwhile.	Reflective Motivation	4.49 (0.74)
I am willing to hire autistic people	Reflective Motivation	4.37 (0.80)
I am willing to adjust the work environment (e.g. office space, lighting) or tasks (activities, processes, etc.) in my organisation to help autistic employees work effectively	Reflective Motivation	4.24 (0.85)
Making adjustments to the hiring process to help autistic applicants perform well will help me to hire the best person for the job	Reflective Motivation	4.20 (0.82)
I am willing to adjust the hiring processes in my organisation to help autistic applicants perform well	Reflective Motivation	4.14 (0.85)
Autistic people have the necessary work skills to be good workers in our organisation	Reflective Motivation	4.11 (0.89)
Hiring autistic people would not decrease my organisation’s performance	Reflective Motivation	4.01 (1.01)
Employing a diverse workforce is part of our organisation’s ethos	Social Opportunity	4.24 (0.92)
It is not fair to the other members of our organisation to make adjustments for autistic people^a^	Social Opportunity	4.21 (1.00)
Our customers/service users would not react negatively to the company employing autistic people	Social Opportunity	4.02 (1.03)
I feel positive about hiring autistic people to work in my organisation	Automatic Motivation	4.01 (0.91)

a Scores for this item have been reverse scored.

Over half of the sample (*N* = 865) provided qualitative data to the four optional open-ended questions: (1) advantages and (2) disadvantages of hiring autistic people, (3) additional barriers and (4) factors that would improve the hiring of autistic people. These were organised under question headings and analysed using content coding. Supplemental Table 2 shows coded responses with frequencies (number of people who mentioned each code). The main advantages of employing autistic people were related to autism-specific skills/insight and improved diversity and inclusivity. Participants also mentioned improved public perception from employing a diverse workforce and advantages for other staff (e.g. skills, knowledge, benefits from adjustments). Disadvantages were related to the extra time, resources and/or training thought necessary to support autistic people, and expectations of negative impacts on workplace and client relationships. Negative beliefs about communication issues, lower productivity, inflexible working and mental health issues (e.g. stress, sensory overload) associated with autistic people were also described. Participants referred to specific work settings and tasks which they thought were unsuitable for autistic workers (e.g. customer facing roles, open plan offices) and recruitment-specific barriers (e.g. the interview process, lack of training for hirers, autism disclosure). Suggested methods for improving the hiring of autistic people were better training for the workforce, adjustments to the recruitment process and additional times and resources to provide support.

### Exploratory analysis of correlations between COM-B items and intentions to hire autistic people

To identify which COM-B items would be most important to target to enable changing hiring intentions, we analysed correlations between the COM-B item scores and intention scores. Correlations are shown in Supplemental Table 3 and demonstrate that all the COM-B items were significantly correlated with intentions to hire autistic people. The strongest correlations were related to feeling positive about hiring autistic people, being willing to make adjustments to the work environment and hiring process and prioritising hiring autistic people. Other items with moderate-strong negative correlations with intentions were around negative beliefs about hiring autistic people (cost/ease of making adjustments, beliefs about reduced organisational performance and autistic people not having the necessary work/communication skills).

## Discussion

The current study explored factors associated with intentions to hire autistic people and perceived barriers to hiring autistic people in a large, nationally representative sample of people with hiring experience in the United Kingdom. The factor most associated with strong intentions to hire autistic people was previous experience of hiring autistic people. Further, participants with higher levels of perceived and actual knowledge of autism and younger and male participants had stronger intentions to hire. Participants from larger organisations had stronger intentions to hire autistic people. However, the contribution of business size was small which means that the findings of this study are applicable across all sizes of organisations.

Analysis of the factors associated with hiring behaviours demonstrated that while people were generally motivated to hire and to make adjustments, there were key barriers around knowledge (e.g. of autism and adjustments), organisational processes (e.g. for making and monitoring adjustments) and strategies (e.g. personal hiring and working practices). Qualitative insights demonstrated that stereotyped beliefs about autistic people (e.g. lower productivity) and the consequences of hiring autistic people (e.g. higher costs) were present in the sample, as was the belief that some roles were unsuitable for autistic people (e.g. customer-facing roles). Understanding key barriers to hiring autistic people is vital to explore why high levels of motivation and generally positive attitudes towards autistic people are not being translated into positive employment outcomes.

The recently published Buckland review (DWP, 2024) provided a number of practical recommendations for improving employment outcomes, which relate to the COM-B components of Psychological Capability (e.g. providing guidance for employers on how to reduce barriers to employment for autistic people and providing models for sharing best practice) as well as Motivational factors (e.g. promoting the benefits of employing autistic people and raising awareness with employers). The findings from the current study support the importance of addressing these Psychological Capability and Motivational factors and highlight the importance of changing actual working practices and processes (Physical Opportunity factors). This study demonstrates that while improving knowledge and motivations are necessary, they need to be underpinned by action and resources within workplaces to effect change.

The knowledge levels in the present study’s sample were comparable to knowledge levels in general population samples in other Western populations (McMahon et al., 2020). Scores on the ASKA were similar to a general population sample in the United States (McMahon et al., 2020) and the mean score for ASK-Q was comparable to a general population sample in Canada (Harrison et al., 2023). This suggests that knowledge levels among hirers are not significantly different to general population samples.

Lack of knowledge about autism has been widely identified as one of the main barriers to employing autistic people, both by employers and by autistic employees (Black et al., 2020; Scott et al., 2018). In line with this, our results make clear the significant role of knowledge on hiring intentions. Higher levels of knowledge (both actual and perceived) were associated with increased intentions to hire, while a lack of knowledge (e.g. knowledge of autism, knowledge about adjustments) was identified as a key barrier experienced by hirers. The Behaviour Change Wheel tool (Michie et al., 2014) suggests that education-based behaviour change techniques can be used to increase knowledge about autism which, in practice, could include general staff training about autism and specific instruction for hirers about how to implement inclusive hiring practices.

The results of the current study indicate that practical guidance about how to adapt the workplace for autistic employees may also be required, both to hiring processes and to workplace environments and processes. Making workplace adjustments has been shown to be vital to support autistic people (Annabi & Locke, 2019; Davies et al., 2023; Ohl et al., 2017; Rashid et al., 2018; Scott et al., 2017). The results of this study show that hirers perceive gaps in knowledge around how to make adjustments in the workplace which may partly explain why high levels of motivation may not be matched by employment outcomes. Participants also described hiring behaviours that do not presently support hiring autistic employees. For example, hirers did not consider whether applicants might be autistic during the hiring process and do not have personal strategies for making or monitoring adjustments. In addition, hirers did not think their organisations had policies and procedures in place for appropriate adjustments. This suggests that employers may also benefit from practical guidelines and policies to underpin more inclusive hiring behaviours. The Behaviour Change Wheel tool (Michie et al., 2014) suggests that Environmental Restructuring can be used to address these barriers. This could include providing resources for employers on adapting workplaces and hiring practices and signposting to guidelines and good practice. Setting and monitoring of goals relating to adjustments could also be facilitated by providing tools to monitor implementation and effectiveness of adjustments in the workplace, and providing checklists which can be used to translate intentions and motivation into practice.

In the present study, perceived knowledge of autism was not associated with actual knowledge of autism, reinforcing previous research which found the same mismatch (e.g. McMahon et al., 2020) and indicating that this is also true for hirers. The existence of stereotyped beliefs in our study sample also suggests that there may be a mismatch between what some people think they know about autism and what they actually know. Both negative and positive beliefs about autistic people were among the factors most strongly correlated with hiring intentions in this study, showing that addressing beliefs is vital. Therefore, support for employers which highlights the positive benefits of employing autistic people as well as challenging negative stereotypes may be well placed to improve employment outcomes.

The Behaviour Change Wheel tool (Michie et al., 2014) suggests techniques such as providing information about the consequences of hiring behaviours (e.g. the consequences of the underemployment of autistic people) and comparing and reframing the outcomes of behaviour (e.g. addressing misconceptions and informing about the benefits of hiring autistic people) may be useful.

The employers in the current study expressed beliefs that certain roles would not be suitable for autistic people, particularly in customer-facing roles or in noisy environments. In contrast to this, some roles (e.g. IT or data-based) can be seen as particularly suited to autistic people’s skills. This is reported in the literature (e.g. Annabi & Locke, 2019; Vogus & Taylor, 2018) and was evident in the qualitative data provided in this study. While this may lead to positive outcomes in certain roles, care must be taken that this does not propagate restrictive stereotypes about the types of jobs autistic people can do or reduce the motivation to make adjustments in other roles. Therefore, addressing negative stereotypes may be particularly important in industries or roles where autistic people could be seen as less well suited (e.g. for managerial roles, where autistic people are typically under-represented, ONS, 2022).

In the current study, the factors which were most associated with intentions to hire autistic people were perceived hiring experience and having a close contact who is autistic. The suggestion that people’s attitudes towards autistic people are impacted by the quantity and quality of their previous contact has been reported elsewhere (Dickter & Burk, 2021; Kim et al., 2023), and more broadly reflects the well-demonstrated effectiveness of intergroup contact as a strategy for reducing prejudice and discrimination (Pettigrew & Tropp, 2006). These findings indicate that intentions are most likely to change through positive interactions with autistic people as opposed to increased knowledge of autism alone. This shows the benefits of focusing on hiring processes as the initial experience of hiring autistic people may act as a catalyst for ongoing intentions to hire, as well as increasing opportunities to interact with autistic people in the workplace for all members of the workforce.

### Limitations and future directions

Recruiting participants from Prolific meant that only registered Prolific users were invited to take part in the study. This sample is unlikely to fully represent the broader population of hirers in the United Kingdom. For example, there was a small overrepresentation of White British, male and London-based populations in the recruited sample, in relation to the general UK population. However, the sample did include participants from a wide range of demographic groups (in terms of age, gender, ethnicity, geographical location), across the populations of the United Kingdom and from a wide range of industries and is well placed to provide a broad range of perspectives on hiring autistic people. As we included a broad range of people with hiring experience, it is also possible that perspectives could differ between sub-groups (e.g. chief executive officers, line managers, human resources professionals). Future studies could look at the barriers we identified from these different perspectives.

Finally, a potential limitation is that the current study centred around intentions to hire autistic people. We know from a large body of literature on the intention–behaviour gap (see Sheeran & Webb, 2016 for a review) that intentions do not always lead to actual behaviour. Therefore, while this study highlights important targets for changing intentions, further study would be required to evaluate how strongly this relates to changing hiring behaviours. Future work could develop and evaluate employment interventions designed around the key barriers identified in this study and using relevant behaviour change techniques to address these barriers (e.g. environmental restructuring, information provision).

## Conclusion

This is the first time that the TDF and COM-B models of behaviour have been applied to understand employment barriers for autistic people from the perspectives of hirers. The insights from the current work are vital for the development of recommendations, policy and practice to address the harmful employment gap for autistic people. This will also allow employers to access a skilled workforce and reduce the social burden of high unemployment among autistic populations.

This study has identified that while motivations to hire autistic people are high and company ethos generally supports diversity in the workplace, participants perceived barriers in relation to knowledge of autism and how to make adjustments for autistic people. They also perceived a lack of practices and policies within the workplace which would support them to hire more autistic people. These gaps in knowledge and opportunity within the workplace need to be addressed to improve outcomes. This could be done via educational interventions, signposting to appropriate resources for employers, sharing good practice, providing adaptable guidelines, checklists and suggestions which can be implemented in the workplace to change and monitor hiring behaviours and sharing positive employment experiences to highlight the benefits of hiring autistic people. Many of these suggestions were recommended in the Buckland review (e.g. sharing good practice, signposting to resources and sharing positive employment stories) and this study provides support for these recommendations based on empirical data and behaviourally grounded theory. This study also highlights the importance of building on existing motivational strengths by emphasising positive beliefs about autistic people and addressing negative beliefs. Attendance to all these factors will be necessary to improve employment rates of autistic people.

## Supplemental Material

sj-docx-1-aut-10.1177_13623613241301493 – Supplemental material for Understanding the barriers to hiring autistic people as perceived by employers in the United Kingdom Supplemental material, sj-docx-1-aut-10.1177_13623613241301493 for Understanding the barriers to hiring autistic people as perceived by employers in the United Kingdom by Marianne Day, Chantelle Wood, Elizabeth Corker and Megan Freeth in Autism

sj-docx-2-aut-10.1177_13623613241301493 – Supplemental material for Understanding the barriers to hiring autistic people as perceived by employers in the United Kingdom Supplemental material, sj-docx-2-aut-10.1177_13623613241301493 for Understanding the barriers to hiring autistic people as perceived by employers in the United Kingdom by Marianne Day, Chantelle Wood, Elizabeth Corker and Megan Freeth in Autism

sj-docx-3-aut-10.1177_13623613241301493 – Supplemental material for Understanding the barriers to hiring autistic people as perceived by employers in the United Kingdom Supplemental material, sj-docx-3-aut-10.1177_13623613241301493 for Understanding the barriers to hiring autistic people as perceived by employers in the United Kingdom by Marianne Day, Chantelle Wood, Elizabeth Corker and Megan Freeth in Autism
