# Supplementary material for: Understanding the barriers to hiring autistic people as perceived by employers in the United Kingdom
Source: Autism. 2024 Dec 4;29(5):1263–74. doi: 10.1177/13623613241301493 (PMC12038075; doi:10.1177/13623613241301493)
Supplement: sj-docx-1-aut-10.1177_13623613241301493 – Supplemental material for Understanding the barriers to hiring autistic people as perceived by employers in the United Kingdom [file sj-docx-1-aut-10.1177_13623613241301493.docx]

| No. | COM-B Item | Sample Mean (*N*=1212) | Standard Deviation |
| --- | --- | --- | --- |
|  | **Psychological capability** Includes knowledge, skills, behavioural regulation, memory, attention, decision processes | | |
| 1. | I know how to make the hiring process more accessible for autistic people | 2.81 | 1.17 |
| 2 | I know enough about autism to hire autistic people | 3.01 | 1.16 |
| 3. | I know how to adjust the work environment (e.g., office space, lighting etc.) or tasks (activities, processes etc.) to help autistic employees work effectively | 3.06 | 1.24 |
| 4. | I am able to communicate effectively with autistic people in my organisation | 3.66 | 0.94 |
| 5. | During the hiring process, I consider whether the applicant could be autistic | 2.92 | 1.16 |
| 8. | I use strategies to monitor how well I adjust hiring practises (e.g., job adverts, interview processes etc.) for autistic people | 2.72 | 1.13 |
| 9.* | I would need to change my current hiring practises to be able to hire autistic people. | 2.84 | 1.18 |
| 10.* | I would need to change the way I work, to make adjustments to the working environment for autistic people. | 2.72 | 1.14 |
| 12. | As part of my role, I adapt the hiring process to ensure it is accommodating for autistic people. | 3.10 | 1.10 |
| 31. | I know about relevant employment law in relation to employing autistic people | 2.85 | 1.26 |
|  | **Physical Opportunity** Includes environmental context/resources | | |
| 6. | My organisation has processes for making adjustments for autistic people during the hiring process (e.g., to job adverts, interview processes etc.) | 3.03 | 1.25 |
| 7. | My organisation has systems and strategies to monitor whether adjustments to hiring processes (e.g., to job adverts, interview processes etc.) are being made for autistic people | 2.89 | 1.22 |
| 34. | Our current hiring processes enable autistic applicants to perform well | 3.20 | 0.98 |
| 35.* | Our current hiring processes could be improved to enable autistic people to perform well. | 2.08 | 0.87 |
| 36. | Our work environment and tasks can be easily adapted for autistic people | 3.60 | 1.01 |
| 37. | Our organisation has policies and procedures in place to support autistic employees to work effectively | 3.19 | 1.16 |
| 38. | My organisation has sufficient time to support autistic applicants to perform well during the hiring process | 3.41 | 1.09 |
| 39. | My organisation has sufficient resources to support autistic applicants to perform well during the hiring process | 3.47 | 1.08 |
|  | **Reflective Motivation** Includes social/professional role, beliefs about capabilities, beliefs about consequences, optimism, intentions, goals. | | |
| 11. | Hiring autistic people is important to me | 3.42 | 0.96 |
| 13. | I feel confident that I could do what is needed to hire autistic people | 3.61 | 1.04 |
| 14. | I feel confident that I could do what is needed to ensure autistic employees perform effectively in the organisation | 3.65 | 1.00 |
| 15. | In my organisation, autistic job applicants would have as much of a chance of being hired as non-autistic applicants | 3.60 | 1.17 |
| 16. | In other organisations, autistic job applicants would have as much of a chance of being hired as non-autistic applicants | 2.81 | 1.02 |
| 17. | I am confident that autistic people would want to work in my organisation | 3.75 | 0.95 |
| 18.* | Hiring autistic people would decrease my organisation’s performance | 4.01 | 1.01 |
| 19. | Autistic people have the necessary work skills to be good workers in our organisation | 4.11 | 0.89 |
| 20.* | Autistic people do not have the communication skills to work effectively in our organisation | 3.75 | 1.04 |
| 21.* | Supporting autistic employees in the organisation would cost my organisation too much money | 3.98 | 0.99 |
| 22.* | Supporting autistic employees in the organisation would take my organisation too much time | 3.83 | 1.07 |
| 24. | I am willing to hire autistic people | 4.37 | 0.80 |
| 25. | I intend to hire autistic people in the future | 3.83 | 0.93 |
| 26. | I am willing to adjust the work environment (e.g., office space, lighting etc.) or tasks (activities, processes etc.) in my organisation to help autistic employees work effectively | 4.24 | 0.85 |
| 27. | I am willing to adjust the hiring processes in my organisation to help autistic applicants perform well | 4.14 | 0.85 |
| 28. | I will hire autistic people in the future | 3.92 | 0.89 |
| 29. | If it led to better performance in my organisation, making adjustments for autistic employees would be worthwhile. | 4.49 | 0.74 |
| 30. | Making adjustments to the hiring process to help autistic applicants perform well will help me to hire the best person for the job | 4.20 | 0.82 |
|  | **Social Opportunity** Includes social influences | | |
| 23.* | Our customers/service users would react negatively to the company employing autistic people | 4.02 | 1.03 |
| 40.* | It isn’t fair to the other members of our organisation to make adjustments for autistic people | 4.21 | 1.00 |
| 41. | Employing a diverse workforce is part of our organisation’s ethos | 4.24 | 0.92 |
| 42. | In my opinion, my organisation has inclusive hiring practises which are suitable for hiring autistic people | 3.61 | 1.07 |
| 43. | In my opinion, the other employees in my organisation support hiring a diverse workforce, including autistic people | 3.94 | 0.91 |
|  | **Automatic Motivation** Includes emotions | | |
| 32. | I feel positive about hiring autistic people to work in my organisation | 4.01 | 0.91 |
| 33.* | I feel worried about hiring autistic people to work in my organisation | 3.72 | 1.12 |

Supplementary Table 1: Means and Standard Deviations (*SD*) for the COM-B items. Questions were answered on a 5-point Likert scale: Strongly Disagree (1) to Strongly Agree (5). *Indicates questions which have been reverse coded. Lower scores indicate items which were perceived as greater barriers to hiring autistic people. Higher scores indicate that items were not perceived as barriers.
