# Supplementary material for: Understanding the barriers to hiring autistic people as perceived by employers in the United Kingdom
Source: Autism. 2024 Dec 4;29(5):1263–74. doi: 10.1177/13623613241301493 (PMC12038075; doi:10.1177/13623613241301493)
Supplement: sj-docx-2-aut-10.1177_13623613241301493 – Supplemental material for Understanding the barriers to hiring autistic people as perceived by employers in the United Kingdom [file sj-docx-2-aut-10.1177_13623613241301493.docx]

| Themes and codes | Frequency |
| --- | --- |
| **Advantages** | **662** |
| Skills and insight specific to autistic people | 406 |
| Diversity and difference in the workforce | 310 |
| Inclusive, equality, representativeness | 172 |
| Skills for specific roles | 64 |
| Improving public perception of organisation, visibility of autistic people within the organisation | 61 |
| Advantages for other staff (e.g., skills, knowledge, adjustments) | 59 |
| **Disadvantages** | **510** |
| Time/resources needed to provide support or make adjustments | 207 |
| Negative impact on workplace relationships | 203 |
| Autistic people’s communication and comprehension skills | 156 |
| Autistic people less productive or able to carry out work tasks | 150 |
| Need for additional training for workforce (e.g., awareness training, specific training for adjustments etc.) | 69 |
| Negative responses from clients/service users | 63 |
| Autistic people’s inflexibility, rigid working style, unable to adapt to workplace tasks | 62 |
| Mental health issues, behavioural problems, stress, overwhelm | 57 |
| Specified unsuitable work settings | 41 |
| **Barriers to employing autistic people** | **200** |
| Lack of priority and awareness in organisations. Organisational ethos, stigma, and discrimination. | 72 |
| Certain roles unsuitable (e.g., customer facing, requiring flexibility) | 51 |
| Recruitment barriers (e.g., the interview process, lack of training from recruiting staff) | 32 |
| Support needs and adjustments are too much or unfair to other workers | 29 |
| Autistic people’s communication barriers and social skills | 26 |
| Conflation with learning disabilities and as a spectrum of issues | 24 |
| Disclosure issues (i.e., not knowing if an applicant/worker is autistic) | 14 |
| **Facilitators for employing autistic people** | **155** |
| More and better training for staff and recruiting staff | 75 |
| Adaptations to interview and recruitment processes and work processes | 49 |
| Additional resources and time to provide necessary support | 26 |
| Greater disclosure of autism status | 14 |
| Better societal awareness | 11 |
| Highlight the benefits of hiring autistic people to organisations | 11 |
| More priority around inclusion in organisations | 7 |
| Job carving (i.e., making roles appropriate for autistic people) | 6 |

Supplementary Table 2: Coded responses to four optional qualitative questions with frequencies ( number of participants who gave responses coded into each category).
