# Supplementary material for: Understanding the barriers to hiring autistic people as perceived by employers in the United Kingdom
Source: Autism. 2024 Dec 4;29(5):1263–74. doi: 10.1177/13623613241301493 (PMC12038075; doi:10.1177/13623613241301493)
Supplement: sj-docx-3-aut-10.1177_13623613241301493 – Supplemental material for Understanding the barriers to hiring autistic people as perceived by employers in the United Kingdom [file sj-docx-3-aut-10.1177_13623613241301493.docx]

| **COM-B items** | **Pearson’s Correlation with Intentions scale** |
| --- | --- |
| 1: I know how to make the hiring process more accessible for autistic people | .359* |
| 2: I know enough about autism to hire autistic people | .421* |
| 3: I know how to adjust the work environment (e.g., office space, lighting etc.) or tasks (activities, processes etc.) to help autistic employees work effectively | .376* |
| 4: I am able to communicate effectively with autistic people in my organisation | .430* |
| 5: During the hiring process, I consider whether the applicant could be autistic | .232* |
| 6: My organisation has processes for making adjustments for autistic people during the hiring process (e.g., to job adverts, interview processes etc.) | .327* |
| 7: My organisation has systems and strategies to monitor whether adjustments to hiring processes (e.g., to job adverts, interview processes etc.) are being made for autistic people | .303* |
| 8: I use strategies to monitor how well I adjust hiring practises (e.g., job adverts, interview processes etc.) for autistic people | .381* |
| 9: I would need to change my current hiring practises to be able to hire autistic people. | .263* |
| 10: I would need to change the way I work, to make adjustments to the working environment for autistic people. | .224* |
| 11: Hiring autistic people is important to me | **.635*** |
| 12: As part of my role, I adapt the hiring process to ensure it is accommodating for autistic people. | .461* |
| 13: I feel confident that I could do what is needed to hire autistic people | .477* |
| 14 I feel confident that I could do what is needed to ensure autistic employees perform effectively in the organisation | .488* |
| 15: In my organisation, autistic job applicants would have as much of a chance of being hired as non-autistic applicants | .451* |
| 16: In other organisations, autistic job applicants would have as much of a chance of being hired as non-autistic applicants | .117* |
| 17: I am confident that autistic people would want to work in my organisation | **.513*** |
| 18: Hiring autistic people would decrease my organisation’s performance | **.562*** |
| 19: Autistic people have the necessary work skills to be good workers in our organisation | **.596*** |
| 20: Autistic people do not have the communication skills to work effectively in our organisation | **.501*** |
| 21: Supporting autistic employees in the organisation would cost my organisation too much money | **.544*** |
| 22: Supporting autistic employees in the organisation would take my organisation too much time | **.556*** |
| 23: Our customers/service users would react negatively to the company employing autistic people | .443* |
| 26: I am willing to adjust the work environment (e.g., office space, lighting etc.) or tasks (activities, processes etc.) in my organisation to help autistic employees work effectively | **.673*** |
| 27: I am willing to adjust the hiring processes in my organisation to help autistic applicants perform well | **.690*** |
| 29: If it led to better performance in my organisation, making adjustments for autistic employees would be worthwhile. | .452* |
| 30: Making adjustments to the hiring process to help autistic applicants perform well will help me to hire the best person for the job | **.625*** |
| 31: I know about relevant employment law in relation to employing autistic people | .281* |
| 32: I feel positive about hiring autistic people to work in my organisation | **.724*** |
| 33: I feel worried about hiring autistic people to work in my organisation | **.547*** |
| 34: Our current hiring processes enable autistic applicants to perform well | .397* |
| 35: Our current hiring processes could be improved to enable autistic people to perform well. | -.255* |
| 36: Our work environment and tasks can be easily adapted for autistic people | **.517*** |
| 37: Our organisation has policies and procedures in place to support autistic employees to work effectively | .384* |
| 38: My organisation has sufficient time to support autistic applicants to perform well during the hiring process | .461* |
| 39: My organisation has sufficient resources to support autistic applicants to perform well during the hiring process | .453* |
| 40: It isn’t fair to the other members of our organisation to make adjustments for autistic people | .462* |
| 41: Employing a diverse workforce is part of our organisation’s ethos | .431* |
| 42: In my opinion, my organisation has inclusive hiring practises which are suitable for hiring autistic people | .416* |
| 43: In my opinion, the other employees in my organisation support hiring a diverse workforce, including autistic people | .498* |

Supplementary Table 3: Pearson’s correlations between COM-B items and intentions to hire autistic people scale.

*Correlation is significant at the 0.01 level (2 tailed). Bolded correlations indicate a moderate-strong correlation (>0.5).
